# Evidence-Based Treatment for HIV-Associated Dementia and Cognitive Impairment: Why So Little?

**DOI:** 10.1371/journal.pctr.0020015

**Published:** 2007-03-30

**Authors:** Ronald J Ellis

## The Abacavir Trial in Context

HIV-associated dementia (HAD) and milder forms of cognitive impairment produce a spectrum of disability that ranges from complete inability to care for oneself to reduced work efficiency and quality of life. HAD is believed to arise from a confluence of adverse effects on neuronal function resulting both from HIV itself and from disturbances in cellular signalling, particularly in the immune system, that further damage neurons. More than two decades after the recognition of HAD as a clinical entity, guidelines for antiretroviral drug treatment in people with HAD and cognitive impairment have yet to be established. The study reported in *PLoS Clinical Trials* by Brew et al. [1] was designed to help develop such guidelines by testing whether adding a single “neuroactive” antiretroviral, abacavir, to an existing regimen would benefit brain function in patients with dementia. Abacavir is a potent inhibitor of HIV reverse transcriptase that interferes with the viral lifecycle and shows reasonably good penetration into central nervous system (CNS) tissues. The trial was historically important because it was done at a pivotal time in the development of antiretroviral therapy, as potent combination drug regimens including protease inhibitors emerged into widespread use in the United States, Europe, and Australia.

The rationale for the study was simple and transparent. It was anticipated that this “CNS active” agent would suppress a potential reservoir of HIV in the central nervous system, where other drugs, especially protease inhibitors, might not be effective. At the time this study was designed, a number of important scientific observations about HAD and its treatment had been made. Zidovudine, the earliest available nucleoside reverse transcriptase inhibitor, seemed to improve the motor functions of people with HAD when given in higher doses than normally used for treatment of systemic HIV disease [2]. Additionally, observational data showed that dementia prevalence in the West dropped after zidovudine became available, suggesting that zidovudine prevented HAD [3,4]. Even so, the burden of mild cognitive impairment in HIV remained substantial [5–7].The trial was historically important because it was done at a pivotal time in the development of antiretroviral therapy.

## What Has Been Learnt Since This Trial Was Conducted?

Although the trial reported here [1] was completed in January 1998, publication was delayed for several years. In the years between completion and publication of the trial, knowledge about the impact of antiretroviral therapy on cognitive impairment in HIV continued to accumulate. New cases of severe dementia became less frequent [8,9], and there was clear evidence of improved cognitive function, even in those with mild impairment [10]. Immunity improved and longevity increased in surviving patients with dementia and mild cognitive impairment. This increase in survival was particularly dramatic for individuals with frank dementia: in the era before highly active antiretroviral therapy (HAART), average survival was about five months, whereas in the HAART era it is close to 40 months [11]. However, many affected individuals did not fully recover their baseline cognitive abilities. Thus, the prevalence of cognitive impairment gradually increased [12] and it became more common in persons with higher CD4 counts [12,13]. Finally, evidence emerged, albeit not unanimous, that cognitive recovery was greatest in those who received antiretroviral medications with better CNS penetration characteristics and in those who fully suppressed viral load in cerebrospinal fluid [14].

## Findings of the Abacavir Trial

In this randomized, double-blind, placebo-controlled trial, 105 HIV-positive patients with HAD who were already receiving combination antiretroviral regimens (“stable background therapy” [SBG]) for at least eight weeks were randomized to treatment with abacavir or placebo for 12 weeks, in addition to their existing regimen. Patients in both treatment arms improved substantially, with the median change in a composite neuropsychological performance index (NPZ) at week 12 exceeding one half of a standard deviation. Improvement was slightly, but not statistically significantly better in the abacavir-treated patients than in those receiving placebo.

## Interpretation, Strengths, and Limitations

A strength of this study is that it is one of very few prospective, randomized, blinded comparative trials for HAD. The study was well-designed and the agent, a newly developed, potent, nucleoside reverse transcriptase inhibitor with favorable pharmacological and tolerability characteristics and predicted good CNS penetration, was of great interest. The targeted patient group was clearly defined and had received relatively little attention in prior antiretroviral treatment studies, offering the potential for this study to have a substantial impact on prescribing practices. However, the study's ability to fulfill its goals was substantially limited by several factors, including rapid new developments in antiviral treatment for HIV.

First, although referred to as “optimal” background therapy, participants' regimens at trial entry were for the most part failing, with virologic success (undetectable plasma viral load) having been achieved in only 23% of individuals in the abacavir arm, and 9% of individuals in the placebo arm. This lack of virologic efficacy of SBG may have reflected a variety of contributing factors, including drug resistance, poor adherence to ART resulting from cognitive impairment, and the relative inexperience of practitioners at that time in applying combination regimens. Additionally, since access to abacavir was restricted at the time the study was being performed, it is possible—even likely—that referral of patients to this study was biased towards those who were failing their existing regimens and therefore in need of a new agent.

Second, although the investigators could not have known it at the time, single agent add-on therapy was subsequently shown to be largely ineffective. Antiretroviral treatment guidelines released after the trial began clearly indicate that, for ART-treated individuals with emerging virologic failure (detectable plasma viral load) and suspected drug resistance, providers should change at least two drugs in the regimen to active agents [15]. Thus the main intervention of this study, adding a single agent to an existing, failing antiretroviral regimen, was in retrospect not a viable treatment strategy.

Virologic suppression from baseline to 12 weeks improved somewhat more in the abacavir group (23% to 46%) than in the placebo arm (9% to 13%). Brew et al. [1] interpret this as demonstration of the virologic efficacy of abacavir add-on therapy. However, the median baseline viral load for the abacavir group (3.7 log_10_ c/ml) was substantially closer to the assay detection limit (2.6 log_10_ c/ml) than for the placebo group (4.6 log_10_ c/ml). By way of contrast, effective, modern combination regimens achieve virologic suppression in more than two thirds of patients over 12 weeks, and frequently produce viral load declines of 2–3 logs [16]. Additional evidence of the limited potency of abacavir versus placebo was provided by the lack of CD4 response in both groups. Individuals with CD4 counts comparable to those in this study frequently show substantial CD4 benefit with effective ART. Since abacavir had a very modest effect on the virus, and virtually none on immunity, its effects on brain function may have been so modest as to elude detection by neuropsychological testing.The abacavir trial has taught us several important lessons.

This issue is of particular importance since most of the individuals in this study had mild or moderate, rather than severe cognitive impairment. While the clinical significance of mild cognitive impairment is clear in relation to Alzheimer disease, where early intervention may prevent or delay the onset of frank dementia, its relationship to dementia in HIV-positive patients is less clear. In HIV, mild cognitive impairment does not predictably progress to dementia. Therefore, to demonstrate efficacy, therapies need to be more potent, more patients need to be studied, or both.

One possible explanation for the lack of difference between the abacavir and placebo patients is that there was a large practice effect in both groups; that is, patients took the same tests repeatedly, giving them the opportunity to better their performance with practice, independent of any improvement in brain function. If practice effects were large in both treatment groups, their magnitude might have obscured any incremental improvement related to the hypothesized beneficial effect of abacavir on brain function. Brew et al. [1] argue that this explanation is unlikely. However, the fact that the tests were administered twice (baseline, week 6) before the time point at which the primary outcome was measured (week 12) gave participants ample opportunity to benefit from practice. Other clinical trials with a similar design have also found substantial improvements within individuals randomized to the placebo arm over the course of the trial [17].

Another potential reason for the lack of superiority of abacavir was that patients in both groups were continuing to experience improvement related to their initial ART (SBG), independent of the study intervention. Similar to practice effects, the argument here is that the magnitude of this improvement in all subjects related to SBG overwhelmed any additional incremental benefit related to abacavir itself. However, if both groups were still benefiting substantially from the introduction of their prior antiretroviral regimens, then it should have been possible to demonstrate a graded effect, with improvements being greatest in those who started their background regimens more recently and least in those whose background regimens had been started more remotely. Unfortunately, information about the duration of therapy prior to study enrollment is not provided in the article, so this possibility cannot be adequately evaluated.

## Implications for Clinical Practice, Research, Health Policy, and the Future of HIV Neurotherapeutics

The abacavir trial has taught us several important lessons. First, since potent, combination systemic ART is available it is no longer acceptable to design trials of single antiretroviral agents. Instead, we need to design trials using combination antiretroviral therapies, and clinical trialists need to anticipate emerging developments in ART and plan for their impact. We need to improve the sensitivity and specificity of methods for measuring neurocognitive change over time. For example, the Reliable Change Index represents a methodologically rigorous means of identifying meaningful neuropsychological performance improvement in individual participants [18]. Scores above a statistically specified confidence interval are interpreted as improvement beyond what would be expected based on normal variability and practice effects. We need to better understand the natural history and interrelationships of dementia and milder forms of neurocognitive impairment. Finally, clinically useful predictive and surrogate markers might help to conduct controlled clinical trials rapidly, and with large enough numbers to impact practice.

## Abbreviations

ART: antiretroviral therapy
central nervous system(CNS) CNS: central nervous system
HAART: highly active antiretroviral therapy
HAD: HIV-associated dementia
SBG: stable background therapy
